# Evaluating Inter- and Intraobserver Agreement on Pectus Carinatum Severity and Treatment Outcomes: A Comparison of Subjective and Objective Assessment Methods

**DOI:** 10.1055/a-2466-6407

**Published:** 2024-12-02

**Authors:** Hendrik van Braak, Sjoerd A. de Beer, Sander Zwaveling, Matthijs W. N. Oomen, L W. Ernest van Heurn, Justin R. de Jong

**Affiliations:** 1 Department of Pediatric Surgery, Amsterdam University Medical Center, Amsterdam, The Netherlands; 2 Department of Surgery, Leiden University Medical Center, Leiden, The Netherlands

**Keywords:** pectus carinatum, intraobserver agreement, interobserver agreement, DCS bracing, Ravitch surgery

## Abstract

**Background** Visual examination is crucial for assessing pectus carinatum (PC) severity and treatment results. This cross-sectional study evaluates the inter- and intraobserver agreement of PC deformities before and after treatment.

**Methods** Observers examined medical photographs of patients before and after treatment. Primary outcome was inter- and intraobserver agreement on esthetic results after treatment. Secondary outcomes included inter- and intraobserver agreement on severity and symmetry before treatment, differences in esthetic results after Ravitch surgery and dynamic compression bracing (DCS bracing), and the impact of scars, age, and treatment duration on esthetic results.

**Results** Medical photographs of 201 patients (aged 4–18) were evaluated by five surgeons and five peers. Surgeons and peers demonstrated inadequate (κ < 0.61) interobserver agreement on esthetic results (κ = 0.26, κ = 0.22), severity of PC (κ = 0.43, κ = 0.38), and symmetry (κ = 0.37, surgeons only). Agreement between surgeons and peers on esthetic results (κ = 0.37) and severity before treatment (κ = 0.54) was similarly inadequate. Surgeons and peers demonstrated inadequate intraobserver agreement on esthetic results (κ = 0.49, κ = 0.34), severity of PC (κ = 0.54, κ = 0.48), and symmetry (κ = 0.60, surgeons only). Deformities treated with Ravitch surgery were perceived as more severe but yielded better results. Peers, unlike surgeons, viewed scars as negatively impacting results. No relationship was found between results after treatment and treatment duration ( *p* = 0.682, *p* = .062) or age ( *p* = 0.205, *p* = .527).

**Conclusions** Subjective assessment of PC severity and esthetic results is inconsistent. Three-dimensional scanning could help standardize treatment completion and aid patients and surgeons in determining treatment completion. The psychosocial effects of scars should be addressed when discussing treatment options.

## Introduction

Pectus carinatum (PC) is a common pediatric condition characterized by overgrowth of costal cartilage which causes protrusion of the sternum and adjacent cartilage. At our institution, patients with PC were previously treated with surgical options like the Ravitch procedure and Abramson procedures. However, noninvasive treatment using dynamic compression system (DCS) bracing has become the preferred treatment. 1 2 3 4 Although treatment outcomes are similar, 5 a significant clinical challenge persists. Surgical outcomes are typically definitive, providing clear correction, but determining endpoints for DCS bracing therapy remains challenging.

The subjective nature of PC's esthetic impact makes determining treatment success challenging. Although objective tools like three-dimensional scanning and the Haller index provide measurable data on the reduction of the chest deformity, 6 7 they do not fully capture the patient's own view of success. At our institution, patients' expectations vary widely; some are satisfied with modest improvement, while others aim for an ideal chest appearance. As a result, even with objective measurements, outcomes ultimately hinge on the patient's own view of success and satisfaction, in mutual agreement with the physician. 5 8 9 Differences in treatment success expectations between patients and physicians can influence treatment decisions, like whether or not to start the retainer phase, and treatment duration, raising questions about consistency in evaluating treatment results. We aimed to assess whether these inconsistencies exist.

The aim of this study was to evaluate the inter- and intraobserver agreement, between surgeons and peers, primarily on esthetic results after treatment and secondarily on severity and symmetry before treatment. Additionally, we assessed differences in average ratings between surgeons and peers and explored the influence of scars, age, and treatment duration on esthetic results.

## Materials and Methods

### Study Design

At time of study conceptualization, we already had a sufficient collection of medical photographs available for analysis. This allowed us to conduct a retrospective cross-sectional study wherein medical photographs of pediatric patients with PC were visually evaluated by reviewers. A METC official waiver of ethical approval (W20_235 # 20.271) was granted from the METC of the Amsterdam Medical Center (Chairperson MSc. O. Harlaar). The need for informed consent was waived.

### Patients and Reviewers

**Fig. 1:**
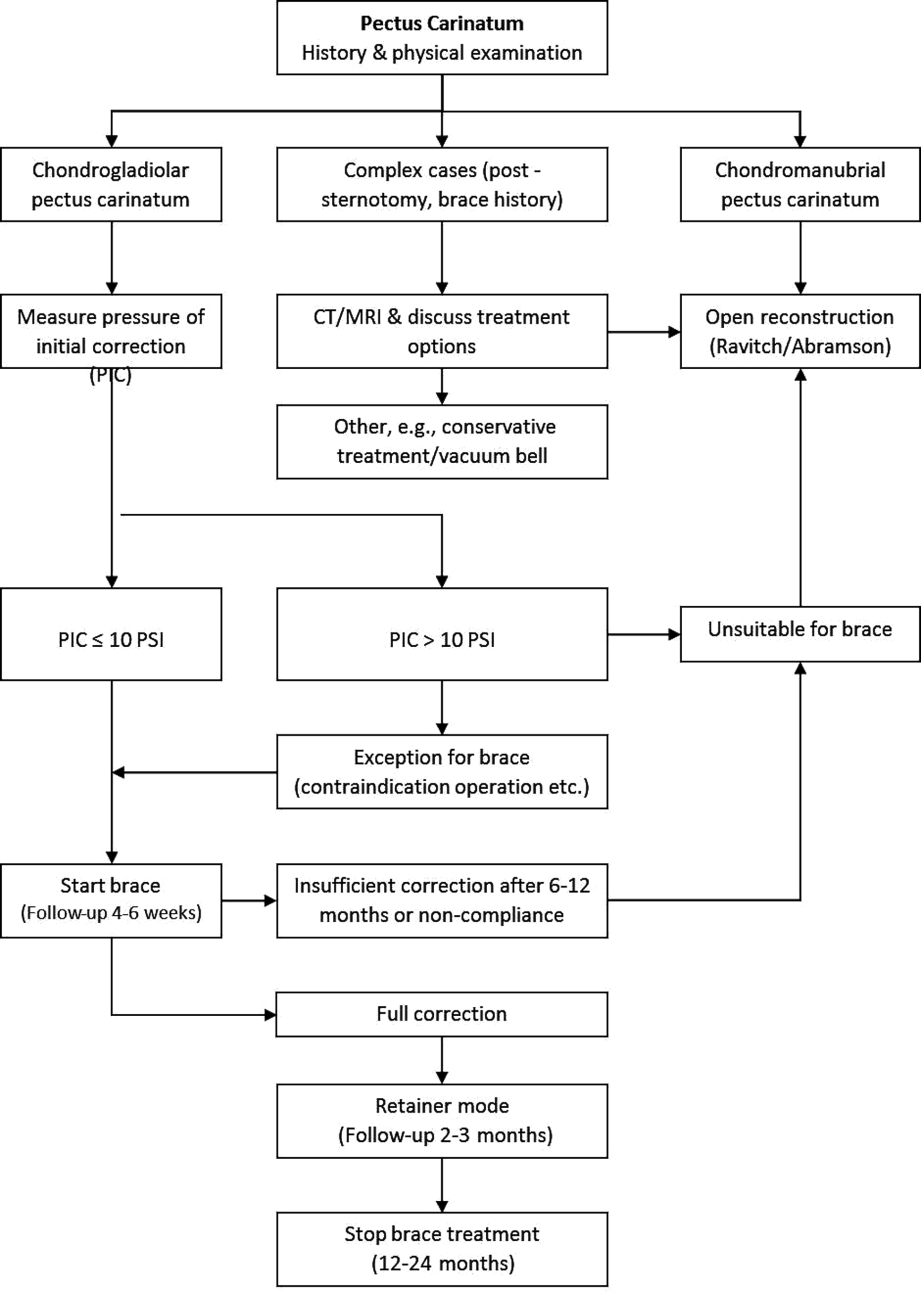
Diagnostic and therapeutic flowchart. CT, computed tomography; MRI, magnetic resonance imaging; PIC, pressure of initial correction; PSI, pounds per square inch.

**Fig. 2:**
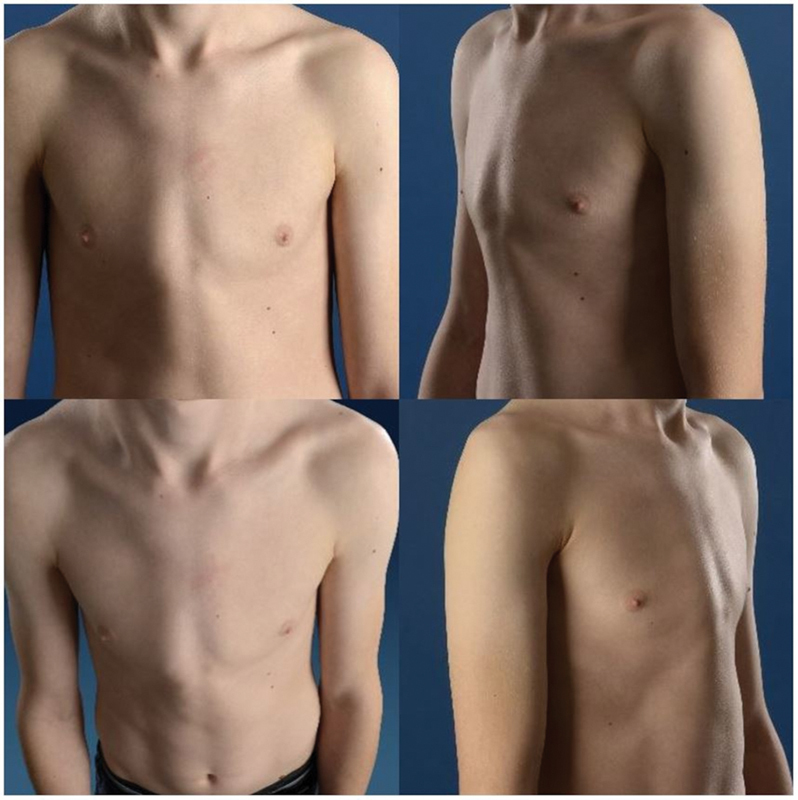
Example of four photographs of a patient before treatment.

**Fig. 3:**
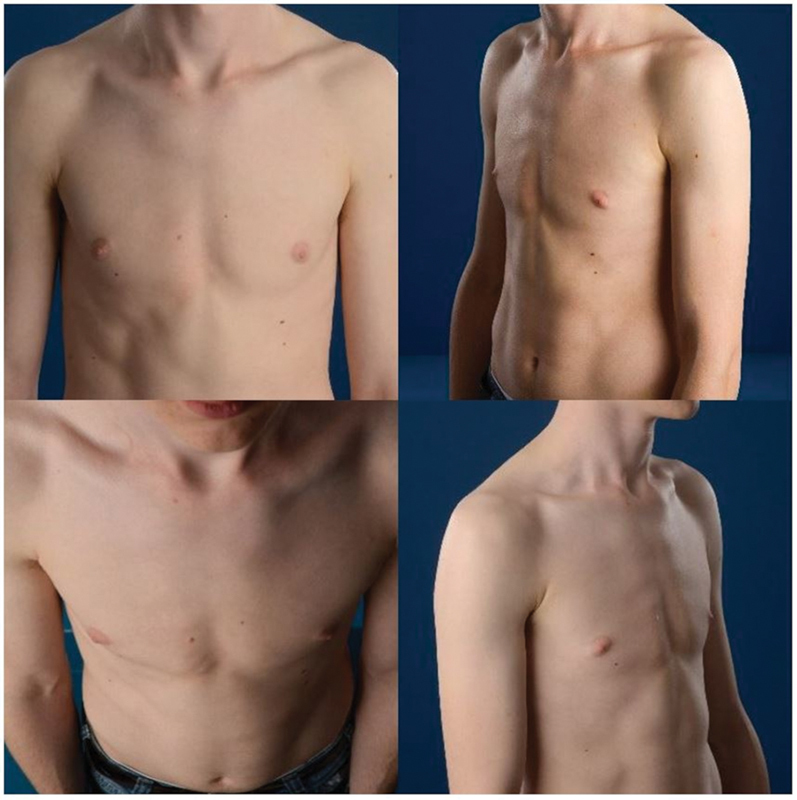
Example of four photographs of a patient after treatment.

All patients who successfully finished treatment for PC with DCS bracing or Ravitch surgery in the Amsterdam Pectus Center between 2009 and 2019, and had before and after treatment photographs taken, were included. Treatment with DCS bracing was regarded successful if patients achieved a satisfactory result, in consultation with the clinician. The results of Ravitch surgery were consistently regarded as successful, as the procedure allows for a precise and complete correction of the deformity. Fig. 1 shows the diagnostic and therapeutic flowchart used in treatment. Eight medical photographs (four before and four after treatment ( Figs. 2 and 3 ) were taken from different angles as part of our standard protocol. Photographs were intended to be taken from every patient, not just the extremes. Photographs were reviewed by five pediatric surgeons and five peers of patients. The limited sample size reflects the exploratory nature of this study in assessing observer agreement on esthetic outcomes.

### Peers

We refer to individuals who share similar age-related characteristics with the patients as peers. Peers were selected instead of patients to provide an external perspective on esthetic outcomes, one that is less influenced by personal treatment experiences or individual biases. These peers were not directly involved in the patients' care nor were they family members and were recruited by posting an open invitation at a local high school and university, asking students to participate in the research. The peers included three males, aged 14, 16, and 23 years, and two females, aged 14 and 16 years. Their age matches the age of patients, to offer a perspective more aligned with the patients' age-related social and esthetic standards, which can be particularly sensitive in adolescent and young adult populations. The number of peers was matched with the number of surgeons to ensure a balanced evaluation, minimizing potential biases from unequal sample sizes.

### Data Collection and Measurements

Data were obtained retrospectively from patient records. Observers were asked to visually examine medical photographs of PC patients and rate the severity before treatment on a three-point scale (one = mild, two = moderate, three = severe) and judge the results after treatment (one = minimal improvement, two = moderate improvement, three = good improvement). Both surgeons and peers were asked to assess esthetic results of patients who underwent surgery with the scar present, and subsequently, visualize the photographs without the scar, providing an extra rating. The scars were not digitally removed; patients were just asked to imagine the photograph without the scar. Furthermore, surgeons were asked to determine if the deformity before treatment was symmetrical or not. This additional question was included exclusively for surgeons, as this factor can influence treatment selection and approach. This question was not posed to peers because they lack the clinical context to understand how symmetry affects treatment decisions, rendering the question redundant. Observers were blinded to all clinical information and completed the assessment twice, with at least 2 weeks interval between both assessments.

### Outcomes

Primary outcomes were interobserver and intraobserver agreement on esthetic results after treatment; secondary outcomes were inter- and intraobserver agreement on severity and symmetry before treatment, differences in average ratings between the Ravitch and DCS bracing groups, and the influence of scars, age, and treatment duration on esthetic results after treatment.

### Statistical Analysis

Descriptive measurements were utilized to characterize the study population. To assess interobserver agreement on esthetic results, severity, and symmetry, we applied Cohen's kappa statistics to all pairs of observers using ratings from the initial assessment round. Cohen's kappa was selected because it accounts for chance agreement, providing a more robust measure of agreement than simple percent agreement. The weighted arithmetic mean of all observer pairs, determined by the number of evaluated subjects, was adopted as a measure of overall agreement. 10 Additionally, the interobserver agreement per observer was based on the weighted arithmetic mean of all observer pairs that included the observer in question. We computed two-sided 95% confidence intervals (CI). A similar approach was taken for intraobserver and scar versus no-scar agreement, but this time, we involved repeated assessments by the same surgeon. Average agreement was based on the mean of all observers. Prevalence-adjusted bias-adjusted kappa (PABAK) was employed to assess agreement when there was substantial disparity in the prevalence of response options. 11 PABAK was specifically chosen as it adjusts for imbalances in response distribution, allowing a clearer understanding of agreement where one response may be more common than others. The interpretation of kappa coefficients was as follows: coefficients ranging from 0.00 to 0.20, 0.21 to 0.40, 0.41 to 0.60, 0.61 to 0.80, and 0.81 to 1.00 corresponded respectively to slight, fair, moderate, substantial, and almost perfect agreement. These ranges provide context for the clinical significance of observed agreement, with higher values indicating more reliable consistency among observers. A kappa value of 0.61 or above is typically considered adequate agreement, indicating a substantial level of consistency beyond chance. 12 We used Spearman's rank correlation to evaluate the statistical relationship between average ratings on severity before treatment and improvement after treatment. Descriptive statistics were used to calculate differences in average ratings. Descriptive statistics were used to analyze the relationship between the treatment duration and age and the results after treatment.

Statistical significance was determined at a probability value of less than 0.05. Data were analyzed using IBM SPSS Statistics 28.0.

## Results

Between September 2009 and December 2019, a total of 736 patients underwent either Ravitch surgery or DCS bracing for PC at the Amsterdam Pectus Center. Among these, 68.6% ( *n* = 505/736) completed treatment successfully. Medical photographs were taken before and after treatment for 201 patients (aged 4–18) out of this group (39.8%, *n* = 201/505). Although protocol dictates photographing all PC patients who finalize treatment, not all attended their medical photography appointments. Both pre- and posttreatment medical photographs are needed to be eligible for evaluation. The majority of included patients were male (96.5%, *n* = 194), with a median age of 15.0 (interquartile range (IQR) 13.5–16.0) years. DCS bracing was the primary treatment for most patients (69.2%, *n* = 139), while the remaining patients underwent Ravitch surgery (30.8%, *n* = 62). Six (9.7%) patients who underwent Ravitch surgery had previously failed DCS bracing treatment. Median age in the Ravitch group was 15.0 (IQR 14.0–16.0) years compared with 14.0 (IQR 13.0–15.0) years in the DCS bracing group ( *p* < 0.001).

### Interobserver Agreement on Severity before Treatment, Symmetry and Results after Treatment

Table 1 shows the results of the interobserver agreement. Overall, agreement was inadequate across all assessments, as all kappa values were below 0.61, indicating less than substantial consistency.

**Table 1:** Interobserver agreement between surgeons and peers

	Severity before treatment		Results after treatment a
	*Severity* a	*Symmetry* a	
**Surgeons**			
Overall agreement	0.43 (0.37–0.49)	0.37 (0.29–0.45)	0.26 (0.21–0.31)
Surgeon A versus B–E	0.35 (0.27–0.42)	0.45 (0.38–0.53)	0.24 (0.16–0.32)
Surgeon B versus A, C–E	0.45 (0.39–0.52)	0.43 (0.36–0.51)	0.36 (0.28–0.44)
Surgeon C versus AB, DE	0.49 (0.43–0.56)	0.31 (0.23–0.40)	0.36 (0.28–0.44)
Surgeon D versus A–C, E	0.42 (0.35–0.49)	0.29 (0.19–0.39)	0.29 (0.20–0.37)
Surgeon E versus A–D	0.47 (0.39–0.54)	0.38 (0.30–0.47)	0.20 (0.14–0.27)
**Peers**			
Overall agreement	0.38 (0.34–0.41)		0.22 (0.19–0.26)
Peer A versus B–E	0.35 (0.27–0.43)		0.23 (0.15–0.31)
Peer B versus A, C–E	0.47 (0.41–0.53)		0.22 (0.14–0.30)
Peer C versus AB, DE	0.39 (0.32–0.46)		0.23 (0.16–0.31)
Peer D versus A–C, E	0.38 (0.31–0.46)		0.30 (0.23–0.37)
Peer E versus A–D	0.44 (0.37–0.51)		0.23 (0.15–0.30)
Surgeons versus peers b	0.54 (0.48–0.60)		0.37 (0.30–0.45)

Notes: ^a^ κ (95% CI).

b Arithmetic mean of surgeons versus arithmetic mean of peers.

Fair agreement was reached among both surgeons and peers (κ = 0.26 [0.21–0.31] and κ = 0.22 [0.19–0.26] respectively) on results after treatment. The overall agreement of severity before treatment among surgeons showed a moderate agreement (κ = 0.43 [0.37–0.49]). This was slightly higher than the fair agreement observed among peers (κ = 0.38 [0.34–0.41]). Surgeons exhibited fair agreement on symmetry (κ = 0.37 [0.29–0.45]). There was a moderate agreement between surgeons and peers on severity before treatment (κ = 0.54 [0.48–0.60]) and a fair agreement on results after treatment (κ = 0.37 [0.30–0.45]).

There was a significant relationship between average rating on severity before treatment and improvement after treatment (surgeons and peers r = 0.39, *p* < 0.001 and r = 0.43, *p* < 0.001 respectively), with more severe deformities resulting in more noticeable improvements.

### Intraobserver Agreement on Severity before Treatment, Symmetry and Results after Treatment

Table 2 shows the results of the intraobserver agreement. Overall, agreement was inadequate across all assessments, as all kappa values were below 0.61, indicating less than substantial consistency.

**Table 2:** Intraobserver agreement of peers and surgeons

	Severity before treatment		Results after treatment a
	*Severity* a	*Symmetry* a	
**Surgeons**			
Average agreement	0.54 (0.47–0.61)	0.60 (0.46–0.74)	0.49 (0.40–0.58)
Surgeon A versus A	0.40 (0.28–0.51)	0.75 (0.66–0.84)	0.50 (0.37–0.63)
Surgeon B versus B	0.55 (0.45–0.64)	0.63 (0.52–0.73)	0.56 (0.46–0.67)
Surgeon C versus C	0.59 (0.41–0.77)	0.71 (0.52–0.89)	0.61 (0.41–0.81)
Surgeon D versus D	0.59 (0.49–0.68)	0.34 (0.21–0.47)	0.34 (0.24–0.45)
Surgeon E versus E	0.55 (0.45–0.65)	0.58 (0.47–0.69)	0.44 (0.34–0.54)
**Peers**			
Average agreement	0.48 (0.38–0.59)		0.34 (0.28–0.40)
Peer A versus A	0.34 (0.23–0.46)		0.22 (0.11–0.34)
Peer B versus B	0.61 (0.53–0.70)		0.36 (0.24–0.47)
Peer C versus C	0.50 (0.40–0.59)		0.40 (0.29–0.50)
Peer D versus D	0.38 (0.28–0.48)		0.34 (0.23–0.44)
Peer E versus E	0.60 (0.51–0.70)		0.37 (0.26–0.37)

Note: ^a^ κ (95% CI).

Surgeons exhibited more consistent results on severity before treatment (κ = 0.54 [0.47–0.61]) and results after treatment (κ = 0.49 [0.40–0.58]) compared with peers (κ = 0.48 [0.38–0.59] and κ = 0.34 [0.28–0.40] respectively). Surgeons showed a moderate average intraobserver agreement (κ = 0.60 [0.46–0.74]) on symmetry.

### Difference in Average Ratings between the Ravitch and DCS Bbracing Groups

Table 3 presents the differences in average ratings between the two treatment groups. Both surgeons and peers consistently rated deformities in patients treated with Ravitch surgery as more severe compared with those treated with DCS bracing. The analysis of the results after treatment reveals a similar trend, with superior results in the Ravitch group compared with the DCS bracing group. On average, peers rated deformities before treatment as more severe than surgeons (2.11 versus 1.91, *p* < 0.001). Peers on the other hand were less positive on the results compared with surgeons (2.23 versus 2.40, *p* < 0.001).

**Table 3:** Average rating of severity before treatment and results after treatment

	Severity before treatment				Results after treatment			
	*Overall*	*DCS bracing*	*Ravitch*	*p-value*	*Overall*	*DCS bracing*	*Ravitch*	*p-value*
Surgeon A	1.67	1.60	1.84	0.016	2.71	2.65	2.94	0.002
Surgeon B	1.83	1.63	2.26	<0.001	2.50	2.37	2.79	<0.001
Surgeon C	1.97	1.76	2.44	<0.001	2.33	2.12	2.79	<0.001
Surgeon D	2.00	1.76	2.56	<0.001	2.51	2.41	2.74	0.004
Surgeon E	2.08	1.91	2.45	<0.001	1.92	1.78	2.21	<0.001
Peer A	2.33	2.22	2.60	<0.001	2.34	2.30	2.44	0.004
Peer B	2.21	2.01	2.66	<0.001	2.33	2.38	2.21	0.006
Peer C	1.84	1.65	2.26	<0.001	2.05	2.01	2.15	0.002
Peer D	1.96	1.83	2.24	0.002	2.33	2.27	2.48	0.020
Peer E	2.20	2.03	2.58	<0.001	2.09	2.03	2.23	<0.001

Abbreviation: DCS bracing, dynamic compression system bracing.

### Influence of Scars, Age, and Treatment Duration on Esthetic Results

All surgeons exhibited an adequate and almost perfect agreement (κ = 0.89 [0.85–0.94]) on results with scars compared with results “imagining no scar,” as shown in Table 4 . This in contrast with patients' peers (κ = 0.39 [0.19–0.60]), exhibiting inadequate agreement.

**Table 4:** Scars versus no scars

	Scars versus no scars
**Surgeons**	
Average agreement	0.89 (0.85–0.94) a
Surgeon A	0.90 (0.81–0.99) b
Surgeon B	0.97 (0.90–1.0) b
Surgeon C	0.90 (0.78–1.0) b
Surgeon D	0.84 (0.70–0.98) b
Surgeon E	0.86 (0.75–0.97) b
**Peers**	
Average agreement	0.39 (0.19–0.60) a
Peer A	0.67 (0.50–0.83) a
Peer B	0.27 (0.14–0.40) a
Peer C	0.62 (0.43–0.80) a
Peer D	0.20 (0.06–0.34) a
Peer E	0.20 (0.09–0.31) a

Notes: ^a^ κ (95% CI).

b Prevalence-adjusted bias-adjusted kappa (PABAK) (95% CI).

There was a significant relationship between age and severity of PC before treatment (surgeons and peers, *p* = 0.010, *p* = 0.029 respectively), but not between age and improvement after treatment (surgeons and peers, *p* = 0.205, *p* = 0.527 respectively).

There was no statistically significant relationship between ratings after treatment and duration of treatment (surgeons and peers, *p* = 0.682, *p* = 0.062 respectively) in the DCS bracing group.

## Discussion

No adequate agreement was reached on esthetic results after treatment, severity of PC, and symmetry. Surgeons exhibited better, but still inadequate, intraobserver agreement compared with peers. Deformities of patients who underwent Ravitch surgery were regarded as more severe, but with better esthetic results afterwards compared with patients who underwent DCS bracing. Peers rated chest deformities more negative than surgeons, assigning higher severity ratings before treatment and indicating less improvement afterward. Age was related to severity of PC before treatment. Both age and treatment duration were not related to final outcomes. More severe deformities before treatment obviously resulted in more noticeable improvements, as opposed to milder deformities.

### Inter- and Intraobserver Agreement on Results after Treatment

Although several studies report that treatment outcomes for PC are determined based on patient satisfaction and mutually agreed-upon observed correction of the deformity, 13 14 15 our findings reveal that there was inadequate agreement on esthetic outcomes post-treatment, with low intraobserver agreement indicating limited consistency among raters. Inconsistent assessment of esthetic outcomes can lead to unclear treatment endpoints, misaligned patient expectations, and challenges in standardizing care. This underscores the need for objective evaluation methods in clinical practice.

A further complication is that developed pectoral muscles and a higher body mass index (BMI) can effectively mask PC. Many patients in our study with “good improvement” developed big pectoral muscles, questioning whether PC actually improved, or was merely concealed by the pectoral muscles. If the results are primarily attributed to changes in muscle mass or BMI, there is a risk of recurrence of esthetic complaints when these factors change again.

Currently, patients are encouraged to build muscle as part of their treatment but are also cautioned that PC could reappear if they lose weight or muscle mass. Therefore, managing expectations before starting treatment is essential. Similar to observations in patients with pectus excavatum (PE) treated with vacuum bell therapy, 16 we find that as patients grow older and reach adulthood, they often become less concerned with their PC and are more likely to accept it if mild recurrence occurs over time.

Thus, an effective approach for obtaining a more objective assessment of PC outcomes should be one that is unbiased, but also capable of adjusting for variables in muscle mass and BMI to ensure accurate assessment of treatment success. Three-dimensional scanning is one such a technique, 6 7 17 18 19 20 21 and has been shown to be able to account for BMI. 22 This method can evaluate the initial severity of PC and, importantly, monitor progress throughout treatment. Over time, it may also serve to establish objective treatment endpoints, helping to create a more standardized approach to patient care.

However, in practice, some patients may feel satisfied earlier than others, regardless of set endpoints. Some patients may wish to end therapy before reaching the set endpoints, while others may prefer to continue beyond them. Despite having determined treatment endpoints, the cosmetic nature of PC means there are no risks associated with shorter or longer treatment durations, making these varying preferences a complication in the treatment process. Additionally, although three-dimensional scanning is user-friendly, it demands extra time from physicians, whose time is already limited.

### Inter- and Intraobserver Agreement on Severity and Symmetry before Treatment

Determining which treatment is suitable for patients is usually decided based on pressure of initial correction (PIC), symmetry, and flaring. When PC is too rigid or when the deformity is too asymmetrical or accompanied by flaring, a surgical correction is technically necessary. 5 Therefore, fair to moderate inter- and intraobserver agreement on PC severity is less of a problem compared with the inconsistency regarding outcomes, since subjective evaluation of severity of PC does ultimately not influence the choice of treatment. Symmetry, on the contrary, does influence the choice of treatment, suggesting that the inadequate agreement on symmetry could have implications.

However, symmetry was not defined prior to the study because in practice also no consistent definition of symmetry was used. This may have led to the different interpretations of symmetry between surgeons. A clear, consistent definition of asymmetry is necessary since it can potentially influence clinical decision-making. An asymmetry analysis as described in the literature could be utilized for this purpose. 17

We found no relationship between severity of PC and treatment duration, likely due to inadequate inter- and intraobserver agreement on severity.

### Ravitch versus DCS Bracing

Deformities in surgically treated patients were consistently rated as more severe than those in patients treated with DCS therapy, by both surgeons and peers. Naturally, more severe deformities yield more noticeable improvements, compared with the milder deformities in the DCS bracing group. This likely explains the better perceived results in the Ravitch group relative to those treated with DCS bracing.

Interestingly, when examining the same data, peers tend to rate both the initial deformities and post-treatment results more negatively than surgeons. This may be due to surgeons' greater familiarity with these deformities, leading to lower severity ratings based on their clinical experience. Additionally, peers may be more sensitive to esthetic factors like scarring, which influences their assessments of post-treatment results more critically than those of surgeons.

### Influence of Scars on Esthetic Results after Treatment

Our results show a significant difference in how surgeons and patients' peers perceive the impact of scars on results after treatment. Surgeons may regard scars as a minor issue due to their focus on achieving structural correction and clinical outcomes, viewing scars as a common, unavoidable aspect of effective surgical intervention. This highlights the importance of considering individual perspectives when counseling patients on treatment options like the Ravitch procedure, and especially the minimally invasive Abramson procedure, as scars can significantly impact patients and are avoided with DCS bracing.

### Age and Severity of Treatment and Influence on Esthetic Results

The relationship between age and severity of deformities before treatment may reflect the age difference between the Ravitch and DCS bracing groups, with the Ravitch group generally being older. Patients in the Ravitch group also had more severe deformities, suggesting that severity might confound the observed association between age and deformity. Although earlier bracing for younger patients could be considered to prevent deformity progression, we view the risk of relapse as a greater concern than progression and therefore prefer to initiate treatment at an older age to minimize this risk. 5 Additionally, age was not found to be related to better esthetic outcomes, suggesting there is no advantage to starting treatment earlier in terms of achieving improved results.

### Influence of Treatment Duration on Esthetic Results

No relationship was found between treatment duration and results after treatment. This result might be biased because all our patients were already regarded as successfully treated.

## Limitations

The fact that only 39.8% of patients had photographs available for analysis raises a concern regarding potential selection bias. Although the reduced sample size may influence the results, there is no clear evidence that patients without photographs differ significantly in outcomes from those included in the analysis, since all patients were treated successfully and were satisfied with the treatment.

A limitation of our study is the potential bias introduced by the difference in experience between the peer and surgeon groups in assessing PC. However, this contrast was intentional, as it mirrors real-world scenarios where patients are inexperienced in evaluation of PC in contrast to surgeons. Understanding how these groups differ in their assessments provides valuable insights into patient experiences and expectations post-treatment. Additionally, the small number of peers reflects the exploratory nature of this experimental study, aimed at gaining preliminary insights rather than achieving broad generalizability.

Despite the impact of flaring on clinical decision-making, a question regarding flaring was not included because surgeons indicated that assessing flaring was not always feasible in our photographs. This would make analysis of flaring in our study unreliable. Although flaring is sometimes considered when choosing between surgery and bracing, its presence or absence does not impact treatment outcomes, which is the primary focus of this study.

In our study we asked observers to visualize the photograph without scar, which may have introduced some bias by placing the burden of imagination on observers and potentially impacting the accuracy of their assessments. A more objective method, such as digitally removing scars from the photographs, could have helped to standardize evaluations and minimize bias by allowing observers to focus solely on the correction of the deformity itself. There may be a difference in visualization skills between peers and surgeons. However, given the clear distinction observed in their assessments, we believe our method proved effective overall.

## Conclusion

Subjective assessments of PC severity and treatment outcomes showed fair to moderate agreement, with peers rating deformities more severely and being more critical of scars. Ravitch surgery led to greater improvements for severe cases, but age and treatment duration had no significant impact on results. The need for objective tools like three-dimensional scanning is clear to improve consistency and treatment evaluation. Future efforts should focus on refining these tools and addressing the psychosocial effects of scars in PC treatment.
